# An osteogenesis mimicking biomineralized bone adhesive for rapid skeletal regeneration

**DOI:** 10.1002/btm2.70182

**Published:** 2026-10-07

**Authors:** Yuqi Liu, Hao Luo, Bin Guo, Xiangming Chen, Martin J. Stoddart, Wen Chen, Xucheng Yang, Yong Zhu, Jianxi Zhu

**Affiliations:** ^1^ Department of Orthopaedics Xiangya Hospital, Central South University Changsha Hunan China; ^2^ AO Research Institute Davos Davos Platz Switzerland; ^3^ National Clinical Research Center for Geriatric Disorders, Xiangya Hospital Central South University Changsha Hunan China; ^4^ Hunan Key Laboratory of Aging Biology Xiangya Hospital, Central South University Changsha Hunan China

**Keywords:** biomimicking, bone adhesive, GelMA, HA, PEGDA, stem cell

## Abstract

Osteogenesis is a crucial process in bone injury repair where osteoblasts mineralize collagen to form new bone at the fracture site. For effective bone regeneration in comminuted fractures, it is essential to develop an osteogenic bone adhesive that can be applied at the time of fixation to temporarily immobilize fragmented bone pieces. We present a flowable, injectable adhesive that enables on‐demand bonding of irregular fracture surfaces and, after photocuring, provides enhanced mechanical integrity while supporting osteogenesis. To meet these clinical requirements, we engineered a gelatin methacryloyl–polyethylene glycol diacrylate (GelMA–PEGDA) network bone adhesive that undergoes rapid photocuring to enable on‐demand bonding and immediate mechanical stabilization at the fracture site. By incorporating nanoscale hydroxyapatite (nHA) to confer a bone‐mineral‐mimetic composition and biomineralization capacity, the photocured adhesive promotes bone marrow‐derived mesenchymal stem cell recruitment and supports skeletal regeneration. During degradation, the organic–inorganic phase of the adhesive can guide osteogenesis, leading to satisfactory healing outcomes in fractures. Overall, the compositional and mechanical biomimicry, photosensitivity and osteogenic potential of GelMA–nHA–PEGDA render it a potential all‐in‐one solution for minimally invasive treatment of multiple skeletal diseases.

**Translational Impact Statement**

We engineered a flowable, injectable gelatin methacryloyl–polyethylene glycol diacrylate bone adhesive that can be applied during fixation to achieve immediate adhesion on irregular, comminuted fracture surfaces and rapidly photocured to enhance mechanical integrity and stabilization. Using our patented photopolymerization delivery system, curing can be performed on demand at the fracture site, enabling practical intraoperative use. Incorporating nanoscale hydroxyapatite provides bone‐mineral–mimetic cues and biomineralization capacity that supports bone marrow‐derived mesenchymal stem cell recruitment and osteogenesis as the adhesive degrades, offering a minimally invasive stabilization‐plus‐regeneration strategy for challenging skeletal injuries.

## 1 INTRODUCTION

Millions of individuals experience complicated bone fractures that need appropriate fixation and repair each year.^1^ Common methods of fixation involve the use of devices such as screws, plates, and pins. However, traditional fixation approaches are accompanied by significant drawbacks including high cost, need of removal, and the risk of infection. These disadvantages have driven the exploration and development of alternative bone regeneration biomaterials.^2^ The concept of bone adhesives has been raised for decades.^3^ Clearly, bone adhesives must possess the capability to establish a robust and enduring bond with bone tissue, producing a mechanically stable interface that can effectively withstand mechanical stress.^4^ Moreover, the ideal bone adhesive should actively promote various processes in bone healing, including recruitment of stem cells into the fracture site, release of key biological factors, deposition of calcium, and bone remodeling.^5^, ^6^

Since the process of natural osteogenesis is biomineralization of collagen, the ideal bone adhesive material should mimic this process.^7^, ^8^ Biomineralization is the process by which cells utilize organic biomolecules as templates to deposit inorganic minerals, resulting in the formation of mineralized structures with high mechanical properties.^9^, ^10^ Bone is a well‐studied system where calcium phosphate biomineralizes collagen fibers. Recently, a biomineralization strategy has been utilized into bone implantation materials.^11^ For artificial biomineralization, utilizing specific proteins as templates to control the biomineralization process and create innovative biomimetic organic/inorganic composites is an intriguing option.^12^, ^13^, ^14^, ^15^ For the organic material of the bone adhesive, Gelatin methacryloyl (GelMA) is an excellent choice for multiple reasons. Firstly, GelMA is derived from gelatin, a partially hydrolyzed form of collagen, and preserves collagen/gelatin‐derived bioactivity, including protease‐mediated degradability (e.g., collagenase‐sensitive cleavage) and cell‐interactive motifs that support cell adhesion and cellular responses.^16^ Additionally, GelMA is a photosensitive hydrogel material that can undergo crosslinking upon exposure to specific Ultraviolet (UV) wavelengths.^17^ GelMA facilitates the connection between osteoblasts and plays an active role in controlling cell polarity, shape, migration, growth, and physiological activities, similar to the function of native extracellular matrix (ECM). Moreover, this hydrogel material is degradable and adapts to the formation of new tissues.^18^, ^19^ Polyethylene glycol diacrylate (PEGDA) hydrogels belong to a crucial group of hydrophilic polymers that exhibit favorable biocompatibility, non‐immunogenicity, non‐toxicity of degradation products, and good malleability.^20^, ^21^ Their internal structure resembles that of the natural ECM of cells.^22^, ^23^ PEGDA hydrogels have been employed in various fields, including biosensors, drug delivery systems, surface modifications, polymerization.^3^, ^24^

Facilitating bone regeneration is the other crucial aspect in the successful use of adhesives for bone tissue repair.^23^, ^25^, ^26^ However, this remains challenging for bone adhesives without weakening the mechanical adhesiveness. Firstly, the proper mechanical strength and porosity are crucial for bone adhesive to facilitate osteogenesis. To effectively guide in vivo osteogenesis, the degradation rate of the adhesive must be properly coordinated with the healing process, while also ensuring sustained regenerative activity.^27^ Specifically, the bone adhesive must degrade at an appropriate rate to permit tissue ingrowth, while also maintaining the ability to induce bone regeneration on the adhesive‐tissue interface throughout the degradation process. Currently, synthetic adhesives such as cyanoacrylate are known to degrade slowly in vivo, which can delay tissue substitution.^28^ Thirdly, the ability of the bone adhesive to recruit stem cells to the fracture healing site is important for stimulating osteogenesis. The natural bone components like collagen and Hydroxyapatite (HA) are reported to stimulate osteogenesis.^29^

In this study, we revolutionize fracture fixation by developing a bone‐matrix‐inspired adhesive that mimics the osteogenic function of osteoblasts to adhere fracture ends and continuously stimulate bone formation. To achieve this, we firstly employed a multi‐functionalization approach using GelMA and PEGDA to form a highly integrated organic–inorganic structure (GelMA–PEGDA). Inspired by osteoblast bone formation processes, we biomineralized the hydroxyapatite nanoparticle (nHA) onto the GelMA–PEGDA network to form the GelMA–nHA–PEGDA organic–inorganic networks, thus strongly increasing the mechanical property and osteogenic potential of the bone adhesives. The flowable GelMA–PEGDA precursor enables minimally invasive delivery into irregular fracture gaps, whereas subsequent photoinitiated radical copolymerization provides rapid in situ solidification and mechanical stabilization. To address in situ curing after injection, our patented photopolymerization delivery system (CN114917011B) delivers 405 nm light via an optical fiber embedded within a guiding sleeve, allowing rapid photocuring directly at the fracture site. The photosensitivity of the GelMA–nHA–PEGDA potentiates adhesives' rapid adherence after injection to the fracture site. Moreover, since the composition and structure of the bone adhesives highly mimics the new bone formed during the regeneration process, the GelMA–nHA–PEGDA shows potential to recruit bone marrow‐derived mesenchymal stem cell (BMSCs) into the fracture site to accelerate bone repair. Since the BMSCs are highly active in the bone adhesives region, the key molecules of active bone regeneration are upregulated (Figure 1d). In sum, the integration of biomimicry, excellent mechanical property, photosensitivity and osteogenic potential of GelMA–nHA–PEGDA renders it a promising all‐in‐one novel solution for the minimally invasive treatment of bone fractures.

**FIGURE 1:**
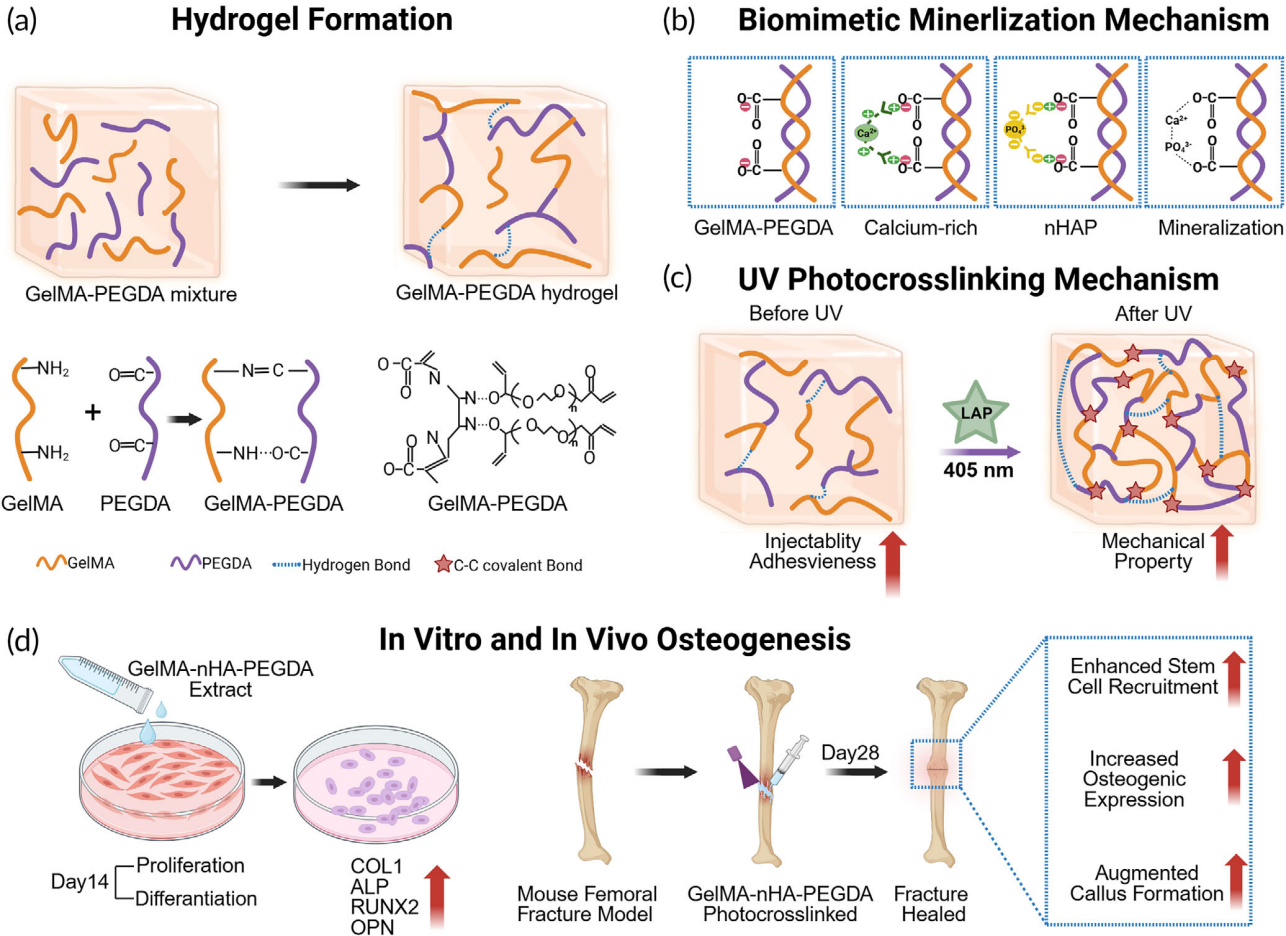
Design rationale and working mechanisms of the gelatin methacryloyl–nanoscale hydroxyapatite–polyethylene glycol diacrylate (GelMA–nHA–PEGDA) bone adhesive, integrating dual‐network formation, biomineralization, and light‐triggered in situ curing. (a) Formation of the GelMA–PEGDA network. Schematic illustrating the crosslinking architecture and network formation between GelMA and PEGDA. (b) Biomineralization pathway. Schematic depicting the mineralization mechanism, including nHA incorporation and the subsequent biomineralization process that establishes a bone‐mineral–mimetic microenvironment. (c) Photocuring process. Illustration of the light‐triggered in situ polymerization workflow, showing adhesive delivery into the defect/fracture gap followed by on‐demand curing under 405 nm light to achieve rapid fixation and mechanical reinforcement. (d) Osteogenic mechanism in vitro and in vivo. Proposed mechanism by which the photocured, mineralized GelMA–nHA–PEGDA adhesive supports osteogenic differentiation and bone regeneration, including recruitment and osteogenic commitment of bone marrow‐derived mesenchymal stem cells and the downstream promotion of new bone formation. LAP, lithium phenyl‐2,4,6‐trimethylbenzoylphosphinate.

## 2 RESULTS AND DISCUSSION

### 2.1 Biomineralization and physical biomimicry of the bone adhesives

To achieve ex vivo mimicry of biomineralization similar to natural bone, calcium‐ and phosphate‐containing components, including calcium nitrate tetrahydrate, diammonium hydrogen phosphate, citric acid, and NaOH, were incorporated directly into the GelMA–PEGDA precursor before photocuring. This process expedited and simulated biomineralization, crucial for creating a bone‐matrix‐inspired, natural bone compositional, and photosensitive glue for rapid fracture repair and skeletal regeneration. The presence of negatively charged carboxyl groups on the GelMA component facilitated the attraction of positively charged ions, particularly calcium ions. This interaction led to the neutralization of phosphate ions, creating conditions conducive to the subsequent binding of calcium ions to the material's surface. The driving force behind this process was the affinity of calcium ions for the negatively charged phosphate surface, acting as a neutral nucleation site. As a result, a balanced interaction allowed the transport of calcium salts and phosphates toward the surface of the hydrogel, triggering the precipitation of hydroxyapatite and concluding the biomineralization process (Figure 1).

To investigate the effects of biomineralization on the material's physical characteristics, various analytical techniques were employed.^3^ Scanning electron microscopy (SEM) was used to examine the morphology of the GelMA–PEGDA hydrogel, which showed a porous structure with irregularly distributed pores ranging from 10 to 100 μm in diameter, and a relatively smooth surface (Figure 2a). After biomineralization, SEM analysis of the GelMA–nHA–PEGDA composite revealed a denser pore structure, with hydroxyapatite nanorods uniformly distributed on the pore walls, while the pore diameter remained in the range of 10–100 μm (Figure 2b). To investigate hydroxyapatite nanorod morphology, transmission electron microscopy (TEM) was employed. The synthesized hydroxyapatite exhibited a nanorod structure, with an average length of 100 ± 19 nm and width of 15 ± 7 nm (Figure 2c). SEM mapping analysis revealed a Ca/P ratio of 1.48 in the sample, closely aligning with the reported Ca/P ratio of 1.50 for hydroxyapatite in the literature,^30^ providing strong evidence for the formation of hydroxyapatite in the studied sample (Figure 2d). Infrared spectrum analysis was conducted to identify the chemical components present. GelMA and PEGDA showed distinct absorption peaks at specific wavenumbers. After biomineralization, new peaks at 600 cm^−1^ and 565 cm^−1^ corresponding to PO_4_^3+^ bending vibration, and at 983 cm^−1^ and 1092 cm^−1^ representing P‐O symmetric and asymmetric stretching vibrations, respectively, were observed. The characteristic peak of the ‐COO‐ functional group in GelMA–nHA–PEGDA (1626 cm^−1^) shifted to a higher wavenumber (1647 cm^−1^) compared to GelMA–PEGDA, indicating the presence of ‐COO‐ in GelMA (Figure 2e). x‐ray diffraction (XRD) analysis further revealed the crystallographic structure of the material. The GelMA–PEGDA hydrogel showed a broad diffraction peak at 2*θ* = 17.5°, suggesting its amorphous nature. However, after biomineralization, distinct and strong diffraction peaks at specific angles (2*θ*) matched the expected positions for hydroxyapatite, confirming its successful formation in the GelMA–nHA–PEGDA composite (Figure 2f).

**FIGURE 2:**
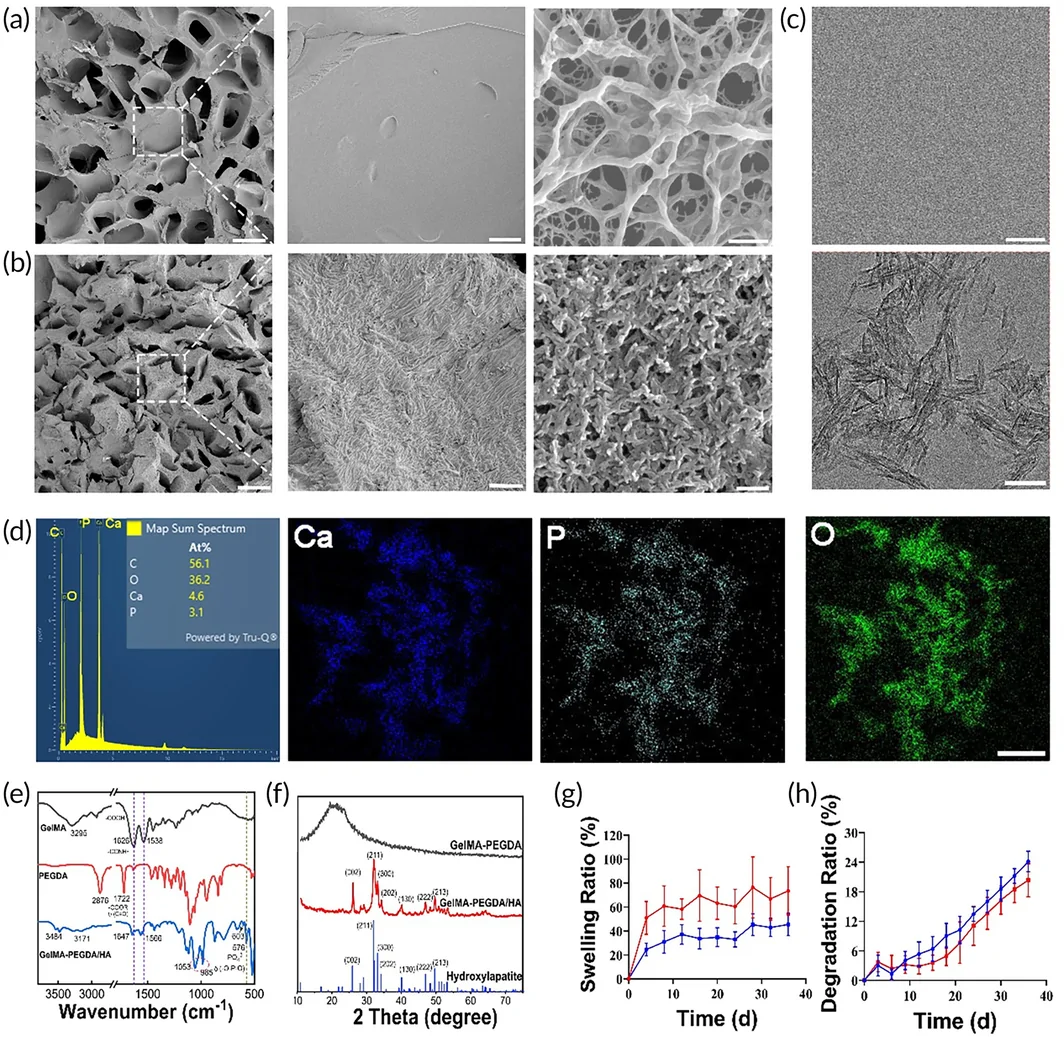
Biomineralization and physical biomimicry of the bone adhesives. (a and b) Scanning electron microscopy images reveal the porous structure of the hydrogel before and after mineralization (bar = 200 μm at low magnification, bar = 20 μm at middle magnification, and bar = 1 μm at high magnification). (c) Transmission electron microscopy analysis confirms the hydroxyapatite crystal structure of the calcium phosphate. The picture above is before mineralization, and the picture below is after mineralization (bar = 100 nm). (d) Scanning electron microscopy with Energy‐Dispersive X‐Ray Spectroscopy (EDS) spot analysis reveals the chemical composition of the hydrogel after mineralization, mainly composed of Ca and phosphate ions (bar = 300 nm). (e) Fourier‐transform infrared spectra of gelatin methacryloyl (GelMA), polyethylene glycol diacrylate (PEGDA), and GelMA–PEGDA/nanoscale hydroxyapatite (nHA), indicating the presence of nHA. (f) x‐ray diffraction results demonstrating the mineralization of PEGDA and GelMA–PEGDA/nHA to form hydroxyapatite. (g and h) The swelling and degradation rate of the bone adhesive in the phosphate‐buffered saline solution of pH 7.4 with or without collagenase type II over time (*n* = 3); *n* = technical replicate.

Collectively, the use of various analytical techniques above allowed for a comprehensive characterization of the physical and chemical properties of the GelMA–nHA–PEGDA composite before and after biomineralization. To assess the water absorption and degradability of the bone adhesive, we conducted a 40‐day immersion of dried bone adhesive specimens in phosphate‐buffered saline (PBS). The swelling and degradation behaviors were evaluated primarily based on the measured mass gain. As shown in Figure 2g, the swelling curve exhibited rapid swelling behavior (>50%) during the initial 3 days, which was attributed to the rapid penetration of water molecules into the bone adhesive and their interaction with the hydrophilic groups, such as ‐OH, leading to the formation of a hydration layer. Subsequently, the bone adhesive nearly reached swelling equilibrium at approximately 25 days, primarily due to the establishment of numerous hydrogen bonds among ‐C=O groups, resulting in a more compact crosslinked network within the bone adhesive (Figure 2g).

Regarding the degradation curve, the degradation rate of the bone adhesive increased progressively with time (Figure 2h). Notably, the bone adhesive group supplemented with type II collagenase showed a higher degradation rate, primarily associated with the degradation of the GelMA component in the bone adhesive. These findings collectively indicate the successful biomineralization of the bone adhesive, together with its in vivo swelling and degradation, highlighting its potential for various biomedical applications.

### 2.2 Adherent and mechanical biomimicry of the bone adhesives

The injectable bone adhesive is a hydrogel designed to interact with biological tissues, serving as both an adhesive and a sealant at the interface between the hydrogel and adjacent tissues.^31^, ^32^ The adhesive properties arise from hydrogen bonding between GelMA and PEGDA, forming a physically associated network that is further reinforced by photoinitiated covalent crosslinking.^33^ Subsequently, biomineralization is applied to deposit nanoscale hydroxyapatite onto the GelMA–PEGDA network, reinforcing its adhesion to bone. At the tissue interface, the carbonyl and ether groups of PEGDA, together with the hydroxyl and amide groups of GelMA, form hydrogen bonds with amino‐ and hydroxyl‐containing groups on the tissue surface. The bone adhesive contains functional groups such as ‐OH, ‐CONH, and ‐NH_2_, which engage in specific hydrogen bonding interactions with corresponding components on the tissue surface, enhancing interfacial adhesion. Additionally, exposure to UV light with a specific wavelength of 405 nm initiates a polymerization reaction between the carbon–carbon double bonds in PEGDA and GelMA, further crosslinking the materials and enhancing their mechanical properties (Figure 1c).

To experimentally evaluate the adhesive capacity of GelMA–nHA–PEGDA, we conducted tests on various materials. It showed strong adhesion to gloves and glass slides, indicating decent adhesive properties (Figure 3a). The bone adhesive exhibited adhesion to various surfaces, including bone, wood, metal, glass, polymers, and other organic materials (Figure 3b). As the adhesive is injectable, it can be easily delivered to bone fracture sites in a minimally invasive manner (Figure 3c). To evaluate its adhesive property, we used fresh cancellous bone pieces from chicken femur and preconditioned them in a PBS solution at 37°C before subjecting them to adhesive tests. The process of bone fracture repair using the adhesive involved injecting it at the fracture site, followed by photocuring with 405 nm light for 20 s (Figure 3d). Once the photocuring process was complete, robust adhesion was achieved between the two fracture sites, withstanding a 500 g weight in vertical orientations (Figure 3e). Moreover, the adhesive property remained stable in a liquid phase in 37°C PBS for 72 h (Figure 3f).

**FIGURE 3:**
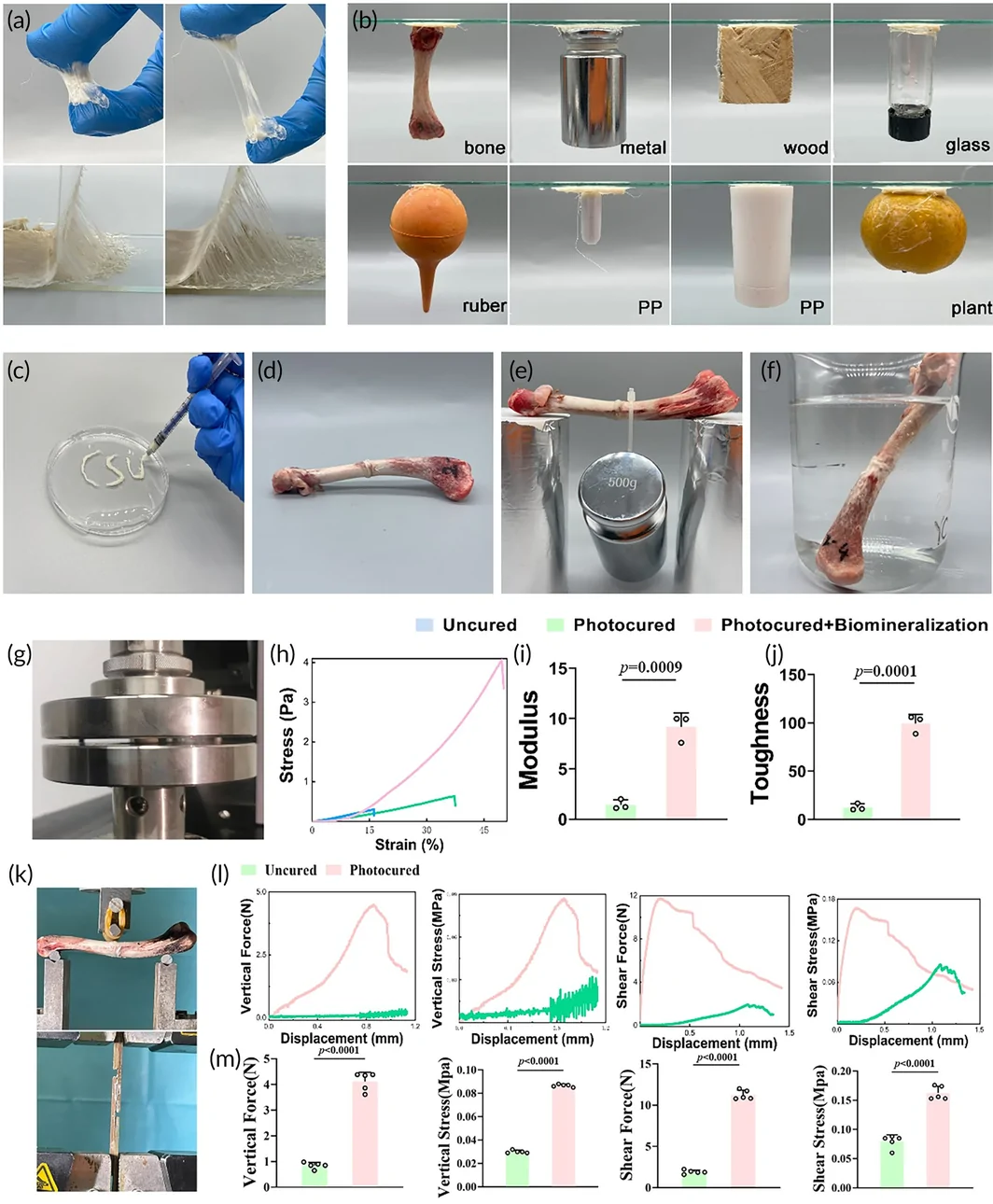
Mechanical biomimicry of the bone adhesives. (a) Macroscopic demonstration showcasing the toughness of bone adhesives under compressive and tensile stress. (b) Photographs displaying bone adhesives adhered to various substrates. (c) Injection of gelled bone adhesives into phosphate‐buffered saline to confirm injectability. (d) Clinical application of bone adhesives and the enhanced mechanical properties after photocuring in a chicken bone. (e) Demonstration of adhesive strength of fractured femoral bones adhered using bone adhesives. (f) Assessment of wet adhesion properties of fresh bone adhesives, demonstrating robustness even after 72 h. (g) Macroscopic and schematic demonstration of lap‐shear tensile and bending tests. (h) Representative pressure‐displacement curves obtained from end‐to‐end compression tests are presented. (i and j) The compression modulus and toughness were determined for mineralized bone adhesives in compression tests and compared to pre‐mineralized bone adhesives (*n* = 3). (k) Macroscopic and schematic demonstration of bending and lap‐shear tensile tests. (l and m) Comparative study between uncured and photocured bone adhesives adhered to fractured femoral bones, recording stress‐displacement and maximum force in lap‐shear tensile and bending tests (*n* = 5), *n* = biological replicate.

To investigate the mechanical strength of the bone adhesive, uniaxial compression tests were conducted to examine the impact of photocuring and biomineralization on enhancing its mechanical properties (Figure 3g). Following photocuring and biomineralization, GelMA–nHA–PEGDA demonstrated a significant increase in maximum pressure prior to failure.

(Figure 3h). Furthermore, biomineralization led to improved mechanical properties under compressive strain, as evidenced by significantly elevated modulus and toughness prior to fracture (Figure 3i,j). Three‐point bending biomechanical tests (testing vertical stress) and lap‐shear mechanical tests (testing shear stress) were also performed, showing substantially higher maximum load and maximum pressure during these tests compared to bone adhesive before photocuring (Figure 3l,m). In summary, biomineralization and photocuring synergistically enhanced the mechanical performance of the adhesive, enabling effective fracture sealing and stable interfacial bonding.

### 2.3 Biocompatibility

Achieving optimal biocompatibility is of utmost importance when designing bone adhesives.^34^ To evaluate the biocompatibility of GelMA–nHA–PEGDA, BMSCs were cultured with material extracts (indirect contact) to assess the effects of soluble components released from the material on cellular behavior. Initially, we tested the dose‐effect relationship by co‐culturing BMSCs with varying concentrations of the material's extracted solution. We observed that BMSCs' CCK‐8 activity increased with the elevation of the extracted solution concentration, reaching its highest level at 75% concentration (Figure 4a,b). Thus, for subsequent biocompatibility tests, we used the 75% material extracted solution. Cell adhesion is another crucial parameter for biocompatibility assessment. In this experiment, we allowed BMSCs to adhere for 1, 4, and 7 h, followed by the removal of non‐adherent cells through washing. Afterward, we added the CCK‐8 reagent and incubated the plate for an additional 2 h. Optical density (OD) values were measured at 450 nm using a microplate reader. The results showed minimal cell adhesion in the Blank group, indicating poor adhesion to uncoated wells. The results showed minimal adhesion‐related signal in the Blank group, consistent with poor cell attachment on uncoated wells. Both GelMA and GelMA–nHA–PEGDA increased OD values relative to the Blank group. Notably, the GelMA–nHA–PEGDA group exhibited significantly higher OD values than the Blank group at 4 h (*p* < 0.001) and 7 h (*p* < 0.0001), whereas no significant difference was observed between GelMA–nHA–PEGDA and GelMA at either time point (Figure 4c). To further test the biosafety of the bone adhesives, we conducted live/dead staining in conjunction with the CCK‐8 assay. The CCK‐8 assay indicated significantly higher OD values in the GelMA–nHA–PEGDA group compared to the control groups, suggesting a high level of material viability (Figure 4d). Moreover, the live/dead staining showed a much higher concentration of living cells (green light), exhibiting an increase in the relative growth rate in the GelMA–nHA–PEGDA group compared to the blank and GelMA group (Figure 4e,f). Taken together, these results collectively suggest that GelMA–nHA–PEGDA exhibits exceptional biocompatibility and safety for in vivo applications. These findings are promising and support the potential use of GelMA–nHA–PEGDA as a bone adhesive with excellent performance in promoting BMSC behavior and overall biosafety.

**FIGURE 4:**
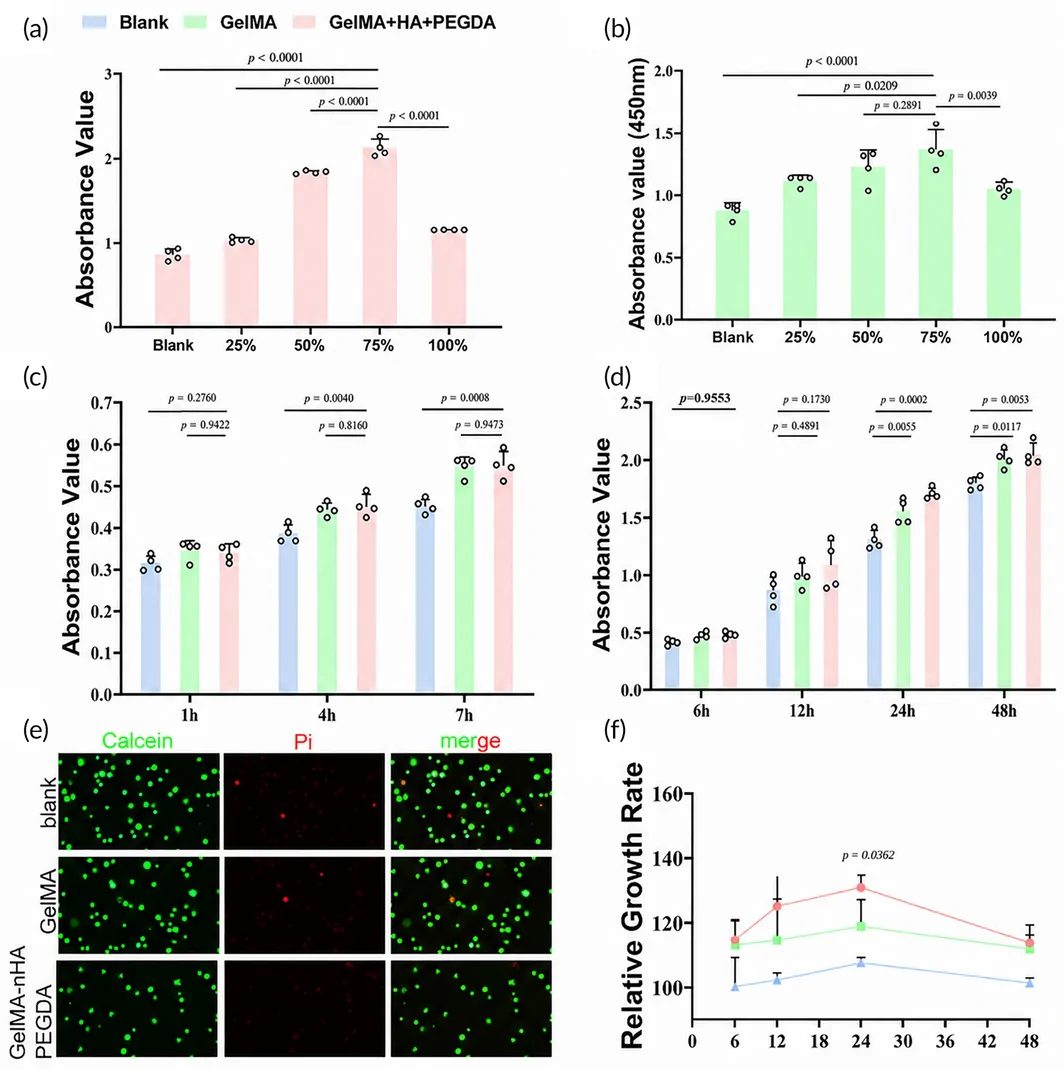
Biocompatibility of the bone adhesives. (a) Cell growth rate of bone marrow‐derived mesenchymal stem cell (BMSCs) in different concentrations of gelatin methacryloyl–nanoscale hydroxyapatite–polyethylene glycol diacrylate (GelMA–nHA–PEGDA) extraction solutions. (b) Cell growth rate of BMSCs in different concentrations of GelMA extraction solutions. (c) Cellular adhesion ability of BMSCs using CCK‐8 assay at 1, 4, and 7 h. (d) Cytotoxicity test of BMSCs using CCK‐8 assay at 6, 12, 24, and 48 h. (e) Representative image of live/dead staining. (f) Relative growth rate of the bone adhesives compared to the control groups (*p* values are indicated in the figure and *n* = 4), *n* = biological replicate.

### 2.4 Osteogenic potential and mechanism of the bone adhesives

Collagen and HA are essential organic and inorganic components of bone, making them crucial for ideal bone adhesive materials that aim to mimic the natural bone composition and provide excellent osteogenic potential. The composition and microstructure of GelMA–nHA–PEGDA closely resemble that of natural bone, providing excellent support and facilitating the secretion of various cytokines by osteoblasts, which promote bone differentiation and growth. To uncover the biological mechanisms underlying the osteogenic properties of the bone adhesive, we first tested its ability to induce BMSC differentiation. As shown in Figure 5a,c, the bone adhesive strongly induced osteogenic differentiation of BMSCs, as evidenced by Alizarin red staining. The staining was more pronounced in the bone adhesive group, indicating enhanced calcium deposition with visible calcium nodules observed compared to the control groups (Figure 5a), further certified by the corresponding statistical analysis (Figure 5c). Additionally, alkaline phosphatase (ALP) is an early differentiation marker of osteoblasts and plays a vital role in regulating the proliferation, migration, and differentiation of osteoblasts. The ALP staining demonstrated that the bone adhesive group had the highest ALP expression compared to the control groups (Figure 5b,d). The study results showed significant differences in the staining intensity of Alizarin Red among the control group, GelMA group, and bone adhesive group (Figure 5c,d).

**FIGURE 5:**
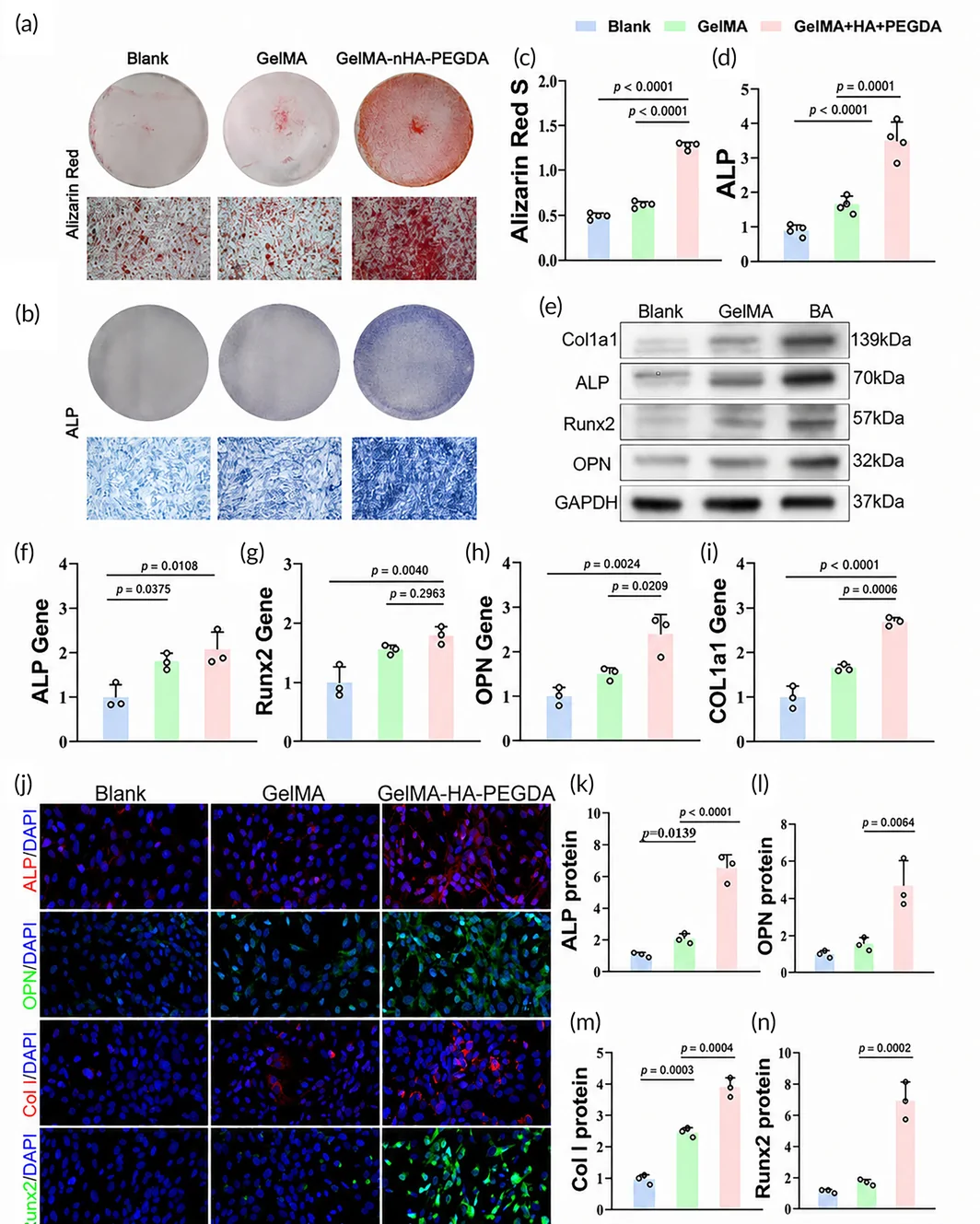
Osteogenic potential and mechanism of the bone adhesives. (a and b) Representative images of Alizarin Red and alkaline phosphatase (ALP) staining results showing calcium nodules. (c and d) Statistical analysis of Alizarin Red and ALP staining activity (*n* = 4). (e) Detection of osteogenesis‐related protein expression in bone marrow‐derived mesenchymal stem cell (BMSCs) cultured in hydrogel by Western blot (ALP, RUNX Family Transcription Factor 2 [RUNX2], osteopontin [OPN], and Collagen1A1 [COL1A1]) (*n* = 3). (f–i) Analysis of osteogenesis‐related gene expression in BMSCs cultured in hydrogel by Reverse Transcription Polymerase Chain Reaction (RT‐PCR) including *ALP*, *RUNX2*, *OPN*, and *COL1A1* (*n* = 3). (j) Representative images of ALP, OPN, COL1A1, and RUNX2 staining of BMSCs treated with bone adhesives or control groups. (k–n) The quantitative assessment of ALP, OPN, COL1A1, and RUNX2 using Image J. *n* = 3, *n* = biological replicate. GelMA, gelatin methacryloyl; nHA, nanoscale hydroxyapatite; PEGDA, polyethylene glycol diacrylate.

To further investigate the molecular mechanisms of the bone adhesive on osteogenesis, we conducted western blot analyses, RT‐PCR, and immunocytochemistry to examine the key osteogenic protein expression. The western blot analysis in Figure 5e revealed that after 14‐day induction, GelMA–nHA–PEGDA significantly improved the protein expression levels of ALP, osteopontin (OPN), Collagen1A1 (Col1A1), and RUNX Family Transcription Factor 2 (RUNX2) compared to the control group. Similarly, the RNA expression levels of *ALP*, *RUNX2*, *OPN*, and *COL1A1* were significantly upregulated in BMSCs treated with GelMA–nHA–PEGDA bone adhesives compared to blank group, as shown in Figure 5f–i. Moreover, immunocytochemistry also demonstrated significantly higher expression of ALP, OPN, COL1A1, and RUNX2 in the bone adhesive group compared to the GelMA group (Figure 5j–n). In conclusion, these findings collectively suggest that GelMA–nHA–PEGDA induces BMSC osteogenic differentiation by stimulating key osteogenic regulatory expressions, including ALP, OPN, COL1A1, and RUNX2. This evidence highlights the bone adhesive's potential as a promising material for promoting osteogenesis and bone regeneration applications.

### 2.5 Skeletal regeneration capability of the bone adhesives

In this study, we evaluated the effectiveness of GelMA–nHA–PEGDA adhesives in fracture repair and bone regeneration using a mouse femoral fracture model. The experimental groups included the unrepaired fracture group as a negative control and the Kirschner needle fixation group as a positive control. For the bone adhesive group, GelMA–nHA–PEGDA adhesives were first applied to adhere to the fracture site, followed by 20 s of UV light exposure for crosslinking. Radiological characterization was performed at 2 and 4 weeks after surgery in live animals using X‐ray films. The unrepaired group showed no healing of the fracture, resulting in the displacement of the fractured ends of the femur. In contrast, both the adhesive group and the Kirschner needle fixation group exhibited healed fractures 4 weeks after the procedure. The x‐ray‐based Lane–Sandhu score demonstrated that the adhesive group had significantly higher scores compared to the unrepaired group at both 2 and 4 weeks after the fracture (Figure 6a,b).

**FIGURE 6:**
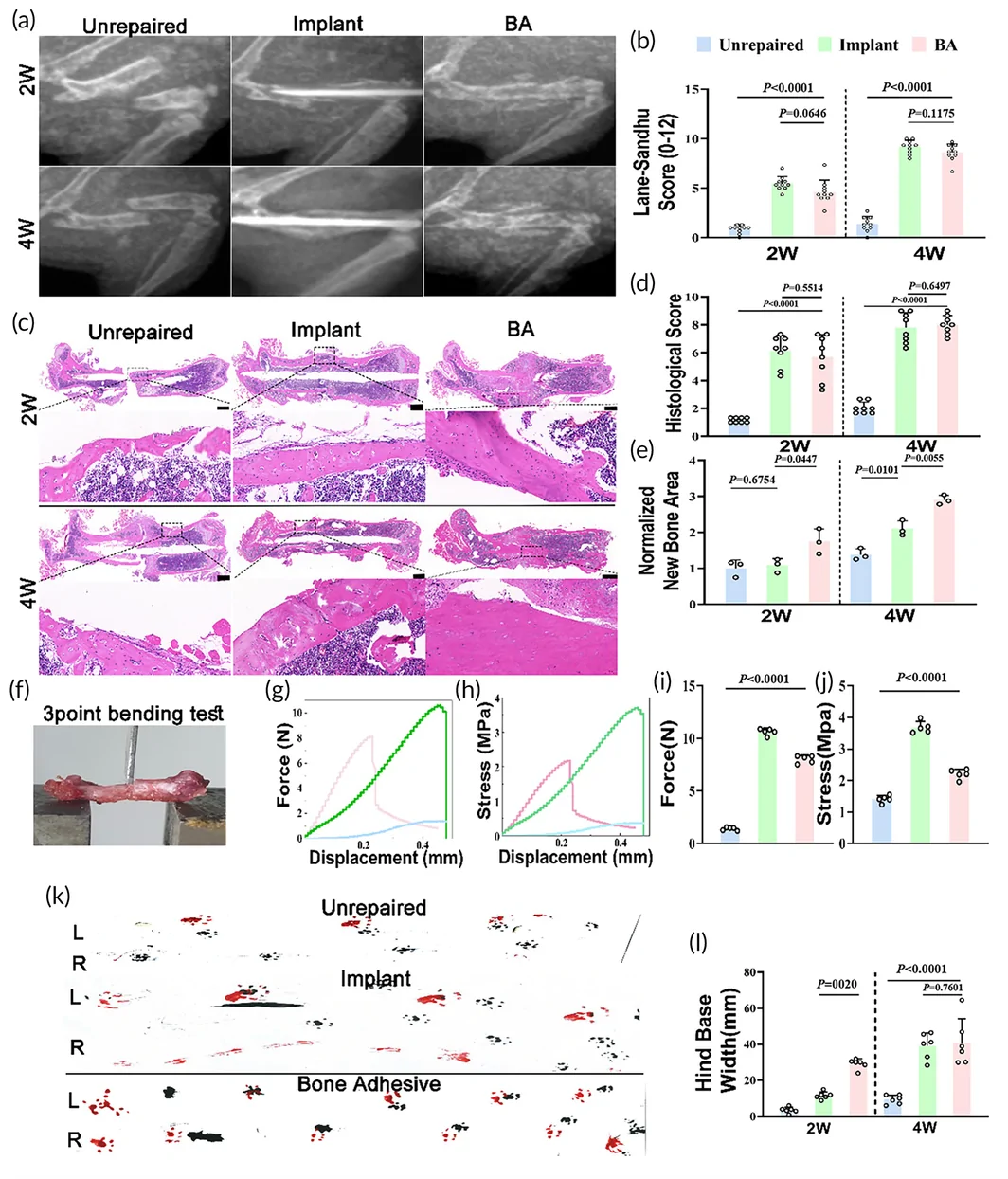
Skeletal regeneration capability of the bone adhesives. (a and b) Representative x‐ray images and statistical analysis of fractured femurs at 2 and 4 weeks post‐surgery, with bone adhesives, implantation, and unrepaired conditions. (*n* = 10). (c–e) Representative hematoxylin and eosin images and quantitative analyses at 2 and 4 weeks post‐surgery (scale bars: 1000 μm, low magnification; 100 μm, high magnification). histological score, *n* = 8 per group; normalized new bone area, *n* = 3 per group. (f–h) Schematic representation, force displacement, and stress curves for the three‐point bending biomechanical test. (i and j) Representative force displacement and stress analysis of femoral samples in the bending test at 4 weeks post‐surgery (*n* = 5). (k and l) Representative footprint images at 2 weeks post‐surgery and quantitative hind base width analysis at 2 and 4 weeks (*n* = 6 per group). *n* = biological replicate.

To investigate the extent of new bone formation in different treatment groups after the fracture, hematoxylin and eosin (H&E) staining was used. Representative H&E images illustrated the histological features of the fracture sites in the three groups (Figure 6c). The GelMA–nHA–PEGDA group exhibited a higher histological score than the unrepaired group (Figure 6d) and the largest new bone area compared with both the unrepaired and Kirschner needle fixation groups (Figure 6e). Furthermore, the mechanical strength of the fracture healing site was measured using 3‐point bending biomechanical tests (Figure 6f). The results showed that both the Kirschner needle fixation group and the GelMA–nHA–PEGDA group exhibited better performance under vertical pressure than the unrepaired group, as evidenced by higher maximal load and maximal stress (Figure 6f–j). Additionally, footprint analysis was used as an indicator of the locomotor proficiency of the mice, with hind base width being a relevant parameter for gait analysis (Figure 6k). The footprints of the adhesive group displayed remarkable clarity, with their hind limbs moving in a wide width. In contrast, the right hind limbs of the unrepaired group and Kirschner needle fixation group mice dragged on the ground, resulting in a smaller hind base width (Figure 6l). In conclusion, GelMA–nHA–PEGDA provided substantially higher immediate normal tensile (0.08 MPa) and lap‐shear strengths (0.15 MPa) than representative fibrin‐based sealants.^35^ Although these values were lower than those reported for some experimental Polyethylene Glycol (PEG)‐based bone glues^36^ and Polymethyl Methacrylate (PMMA)–bone interfaces,^37^ adhesive‐only treatment achieved radiographic fracture healing at 4 weeks and a repaired construct failure stress of approximately 4 MPa, supporting its potential as a standalone alternative to internal fixation in selected fractures.

### 2.6 Mechanism of skeletal regeneration of the bone adhesives

To investigate the biological mechanism underlying the osteogenesis of GelMA–nHA–PEGDA bone adhesives, we conducted micro‐CT analysis to evaluate their effect on bone microstructure. The Three‐Dimensional (3D) reconstruction images clearly demonstrated that the fracture was not healed in the unrepaired group, while complete healing was observed in both the K‐wire fixation group and the bone adhesives group. Notably, the bone adhesives group exhibited evident new bone formation (Figure 7a). By analyzing the new bone area in the different treatment groups, we observed that the GelMA–nHA–PEGDA adhesives group had significantly higher bone surface (BS) and bone volume (BV) (Figure 7b,c). Trabecular bone analysis further revealed significantly higher trabecular thickness (Tb.Th), trabecular number (Tb.N), bone volume/total volume (BV/TV), and less trabecular separation (Tb.Sp) in the bone adhesives group compared to the unrepaired and K‐wire fixation groups (Figure 7d–g). These findings collectively indicate that the bone adhesives group exhibited superior bone formation and density.

**FIGURE 7:**
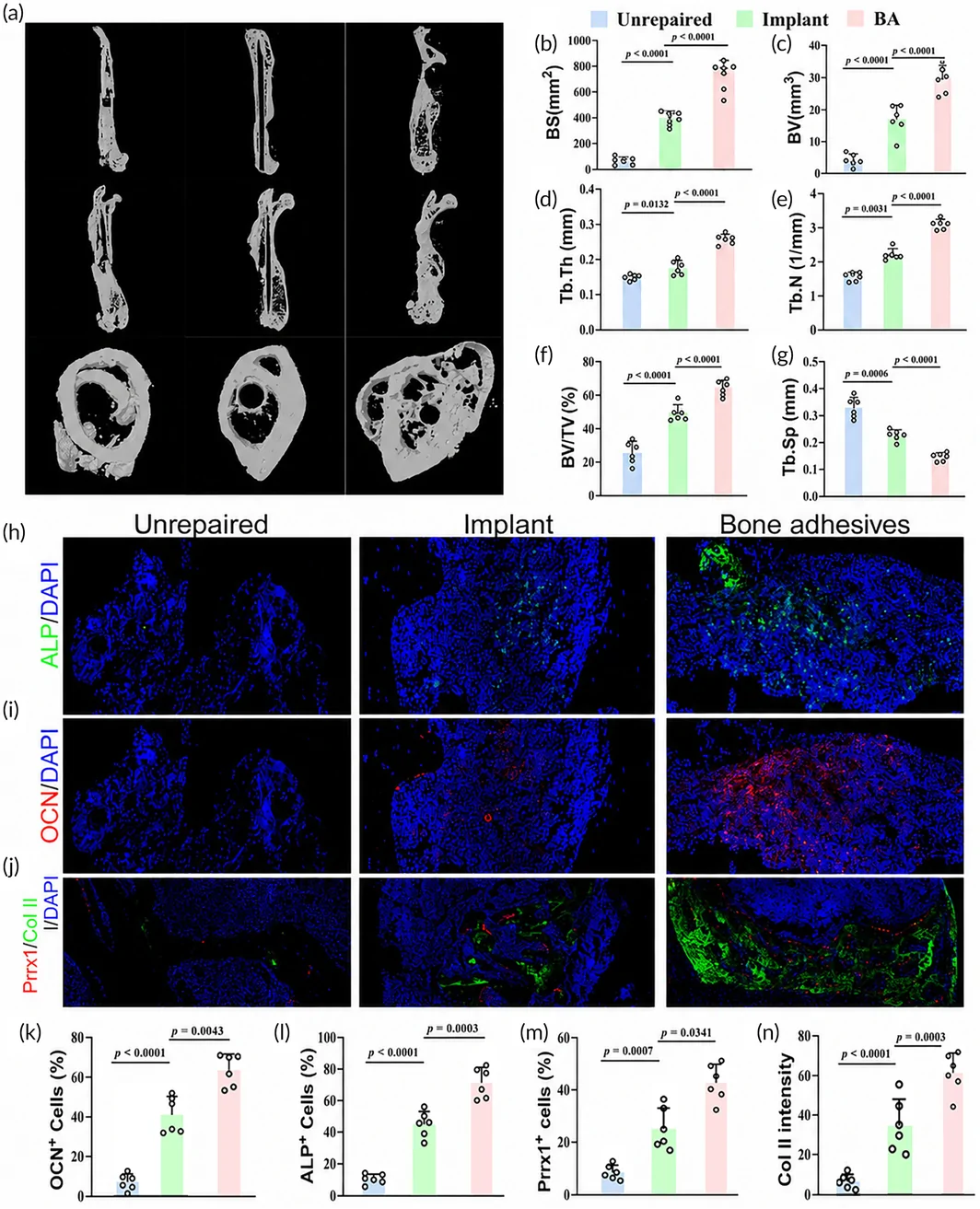
Mechanism of skeletal regeneration of the bone adhesives. (a) Micro‐CT images of transverse, sagittal, and coronal sections of fractured femurs 4 weeks after surgery. (b–g) Quantitative assessment of bone surface (BS), bone volume (BV), trabecular thickness (Tb.Th), trabecular number (Tb.N), bone volume fraction (BV/TV), and trabecular spacing (Tb.Sp) at the fracture sites. (h–j) Representative images of immunofluorescent staining for alkaline phosphatase (ALP), osteocalcin (OCN), paired related homeobox 1 (Prrx1), and type II collagen (COLII) at the fracture sites 4 weeks after surgery. (k–n) Statistical analysis of immunofluorescent staining for ALP, OCN, Prrx1, and COLII at the fracture sites 4 weeks after surgery (*n* = 6), *n* = biological replicate.

Moreover, to investigate the biological mechanism of osteogenesis induced by the bone adhesive, we tested the expression of key factors involved in osteogenesis and stem cell recruitment. The bone adhesives group showed significantly higher expression levels of ALP and osteocalcin (OCN) compared to the unrepaired and K‐wire fixation groups, indicating its better ability to induce osteogenesis at the fracture site (Figure 7h,i,k,l). Additionally, we assessed the stem cell recruitment ability of the bone adhesives by performing paired related homeobox 1 (Prrx1) staining (a stem cell marker). Importantly, the biomineralized GelMA within the bone adhesives was found to be particularly effective in recruiting stem cells, leading to a much better stem cell recruitment ability in the bone adhesives group (Figure 7j,m). Furthermore, collagen II staining was conducted to assess the deposition of the ECM after the fracture. The bone adhesives group exhibited significantly higher collagen II deposition compared to the control groups (Figure 7j,n), indicating enhanced ECM formation and bone tissue organization. Taken together, these results collectively demonstrate that GelMA–nHA–PEGDA adhesives recruit stem cells to the fracture site, where they secrete key osteogenic factors, thereby accelerating the bone regeneration and fracture healing processes. The bone adhesives promote new bone formation, improve bone microstructure, and enhance osteogenic capabilities, making them a promising candidate for facilitating bone regeneration and repair in clinical applications.

## 3 CONCLUSION

In this study, our findings demonstrate that the photocrosslinked GelMA–nHA–PEGDA bone adhesives exhibit favorable mechanical performance under the tested conditions. Firstly, photocuring increased its mechanical stability, and the adhesive showed the ability to bond to various substrates. Secondly, the bone adhesives promote BMSC proliferation and osteogenic differentiation, showcasing a high level of biocompatibility. Co‐culture with the bone adhesives revealed excellent biocompatibility. Moreover, osteogenic properties were evaluated using ALP staining, alizarin red staining, RT‐PCR, and Western Blotting (WB) assays, which confirmed the in vitro osteogenic potential of the bone adhesive. Thirdly, in vivo osseointegration tests demonstrated that GelMA–nHA–PEGDA facilitated rapid adhesion and induced new bone formation, indicating its potential for clinical applications. Therefore, GelMA–nHA–PEGDA bone adhesive holds significant promise for clinical fracture repair and offers substantial advantages in the field of bone tissue engineering.

## 4 MATERIALS AND METHODS

### 4.1 Ethics statement and animal handling

C57BL/6 mice (8 weeks, specific pathogen‐free [SPF]) were obtained from the Department of Laboratory Animal, Central South University (Changsha, Hunan, China). A total of 67 mice were used, comprising 36 females and 31 males. Four mice were used for primary BMSC isolation and were not included in the in vivo comparisons. The remaining 63 mice were allocated to the unrepaired, implant, and Bone Adhesive (BA) groups, with 21 mice per group. Male and female mice were housed separately, with six animals per cage (IVC system) at 22 ± 2°C and 60% relative humidity on a 12 h light/12 h dark cycle, with autoclaved chow and sterile water provided ad libitum. Animals were acclimatized 1 day prior to experimentation. Each mouse was assigned a unique ear‐tag number and treated as one experimental unit. Group allocation was generated using a computer‐based random‐number sequence and was not stratified by sex. Ear‐tag numbers were used throughout surgery, follow‐up, and sample collection. The study was not powered to assess sex‐specific treatment effects. All animal procedures were conducted in accordance with the Chinese National Standard GB/T 35892‐2018 and were approved by the Experimental Animal Ethics Committee of the Department of Laboratory Animal, Central South University (approved animal protocol No. CSU‐2022‐0288).

### 4.2 Fabrication of hydrogel

Methacrylated gelatin (GelMA; Sigma‐Aldrich/Merck, Cat. No. 900629) was used to prepare the photocurable precursor together with calcium nitrate tetrahydrate (Ca(NO_3_)_2_ · 4H_2_O; Sigma‐Aldrich/Merck, Cat. No. C1396), citric acid (anhydrous; Sigma‐Aldrich/Merck, Cat. No. C0759), diammonium hydrogen phosphate ((NH_4_)_2_HPO_4_; Merck, Cat. No. 1.01207), poly(ethylene glycol) diacrylate (PEGDA; average Mn 700; Sigma‐Aldrich/Merck, Cat. No. 455008), lithium phenyl‐2,4,6‐trimethylbenzoylphosphinate (LAP; CAS 85073‐19‐4; Sigma‐Aldrich/Merck, Cat. No. 900889), and sodium hydroxide (NaOH; Sigma‐Aldrich/Merck, Cat. No. S5881), dissolved in sterile deionized water or PBS (pH 7.4; Gibco, Cat. No. 10010023). Briefly, GelMA (2.00 g) was warmed in a hot water bath until fully molten and homogeneous, followed by sequential addition of 2.36 g Ca(NO_3_)_2_ · 4H_2_O (from 5 M stock), 0.31 g citric acid (from 6 M stock), and 0.79 g (NH_4_)_2_HPO_4_ (from 3 M stock), then 0.04 g LAP (added as 2% w/v stock) and 1.20 g PEGDA; the pH was adjusted to 7.0–7.8 by adding 0.06 g NaOH (from 6 M stock), yielding a formulation with an overall mass ratio (GelMA:Ca(NO_3_)_2_ · 4H_2_O:citric acid:(NH_4_)_2_HPO_4_:LAP:NaOH:PEGDA) of 400:236:31:79:4:6:120. Immediately prior to use, the sealed container was pre‐warmed in a hot water bath to restore a fully molten, easily applicable state, and curing was performed by irradiation for 20 s using a portable 405 nm light source (Model EFL‐LS‐1600‐405, EFL factory; nominal output power 3 W; effective illumination diameter 2.5 cm).

### 4.3 Mechanical properties of hydrogel

All mechanical tests were performed using an electromechanical universal testing system (MTS Insight 23, MTS Systems, Eden Prairie, MN, USA) with a calibrated load cell (stated accuracy ±0.5%). Under wet conditions at 25°C, hydrogels were tested in uniaxial compression at 0.5 mm/min to obtain stress–strain curves. The uncured, photocured‐unmineralized, and photocured‐mineralized conditions were assessed as separate groups; compressive stress and modulus were calculated from the corresponding curves (*n* = 3 independent specimens per condition). In vitro adhesive performance was evaluated using a chicken‐femur three‐point bending model and a tensile/lap‐shear bonding model, with loading to failure under monotonic conditions. Dynamic or cyclic loading was not assessed. Static PBS immersion of bonded chicken‐bone fragments for 24, 48, and 72 h was used only as a qualitative attachment demonstration; no load was applied during immersion and adhesion strength was not measured afterward.

### 4.4 Physical characterization test

Adhesive/mechanical performance was evaluated using two in vitro models (a chicken‐bone three‐point bending model and a steel‐to‐steel tensile bonding model) and an in vivo mouse femoral fracture model. For the chicken‐bone model, fresh chicken femurs were cleaned of soft tissue, kept hydrated in PBS, and repaired with the photocurable bone adhesive; specimens were then tested in three‐point bending with a 60 mm support span at a crosshead speed of 0.5 mm/min. The geometric parameters of each femur (outer diameter and cross‐sectional area at the loading region) were recorded and used for normalization of mechanical outcomes, consistent with prior poultry bone biomechanics testing where cross‐sectional geometry is incorporated into strength calculations. For the steel‐to‐steel model, the adhesive precursor was dispensed between two stainless‐steel plates (2 cm × 2 cm) to define a bonded area of 4 cm^2^, and curing was induced by 405 nm irradiation for 20 s; the bonded joints were then loaded in uniaxial tension until failure. Bonded area (*A*) for stress calculations was defined by the measured substrate cross‐sectional area for bone specimens (mouse femur or chicken femur, depending on the test) and by the plate overlap area for the steel‐to‐steel joints; maximum stress was calculated as *σ*_max = *F*_max/*A*. For mouse femurs, cross‐sectional cortical geometry can be defined using mid‐diaphyseal cross‐sectional analysis (e.g., cortical area) as commonly reported in murine femoral mechanics studies. Mechanical testing was performed on an electromechanical universal testing system (MTS Insight 23, MTS Systems, Eden Prairie, MN, USA; force capacity 1 N–30 kN, standard length configuration), with force measured using a calibrated load cell with a stated accuracy of ±0.5%. For the in vivo assessment, a mouse femoral fracture model was established and femurs were harvested at 2 and 4 weeks from three groups: untreated fracture, fracture + internal fixation, and fracture + bone adhesive. Harvested femurs were kept hydrated and mechanically tested to obtain vertical force and displacement under axial loading, enabling comparison of structural stability across treatments and time points.

### 4.5 Isolation of BMSCs

Primary BMSCs were isolated from femurs and tibias of four 8‐week‐old C57BL/6 mice. Mice were euthanized by cervical dislocation, immersed in 75% ethanol, and femurs and tibias were aseptically harvested, cleaned of surrounding soft tissue, and kept in sterile PBS. The epiphyses were removed and the marrow cavities were flushed with PBS using a 1 mL syringe until the bones turned pale; the flush was passed through a 40 μm cell strainer into a 15 mL tube, centrifuged at 1000 rpm for 5 min, and the pellet was resuspended in Mesenchymal Stem Cell (MSC) expansion medium consisting of Minimum Essential Medium alpha (α‐MEM, Thermo Fisher Scientific) supplemented with 10% fetal bovine serum (Sera Plus, Pan Biotech, P30‐3702), 100 U/mL penicillin and 100 μg/mL streptomycin (Thermo Fisher Scientific).

### 4.6 Cell cytotoxicity assay

For extract‐based exposure, GelMA–nHA–PEGDA and the GelMA control were sterilized and aliquoted into 15 mL centrifuge tubes; after accounting for the additional liquid absorbed by the materials, extracts were prepared at 0.2 g/mL in MSC expansion medium (HA powder was also extracted at 0.2 g/mL), incubated on a 37°C shaker for 24 h, and the resulting 100% (original) extracts were collected for cell testing. BMSCs were then cultured with different material extracts, and cell adhesion/proliferation and cytotoxicity were assessed by CCK‐8 (Abcam, ab228554) at the indicated time points (e.g., 1, 4, and 7 h or 6, 12, 24, and 48 h). Briefly, each well received 10 μL CCK‐8 reagent plus 100 μL fresh medium and was incubated at 37°C for 3 h protected from light; the supernatant was transferred to a 96‐well plate and absorbance at 450 nm was measured using a microplate reader (SpectraMax ABS, Molecular Devices, USA).

### 4.7 Live/dead cell staining

Adhesive extracts were prepared in 24‐well plates according to ISO 10993‐12 principles. Briefly, adhesive‐containing hydrogels were incubated in α‐MEM for 24 h at 37°C, and the extracts were collected for subsequent cell testing. Hydrogel surfaces were washed twice with sterile PBS and twice with α‐MEM, then BMSCs were seeded at 2.0 × 10^4^ cells/well and cultured at 37°C, 5% CO_2_. Cell viability was assessed at 2, 6, 12, and 24 h using Calcein‐AM/propidium iodide (PI) staining. Calcein‐AM (Thermo Fisher Scientific, C3099) and PI (Thermo Fisher Scientific, P3566) were diluted in assay buffer (5 mL) to prepare the working solution (5 μL Calcein‐AM + 15 μL PI). At each time point, medium was removed, samples were washed twice with assay buffer, and 100 μL staining solution was added per well followed by incubation for 30 min at 37°C in the dark. Images were acquired using a fluorescence microscope (Leica DMI6000B, Leica Microsystems). Live (green) and dead (red) cells were quantified with ImageJ.

### 4.8 Osteogenesis of BMSCs

Lyophilized materials (GelMA; GelMA+nHA + PEGDA) were sterilized by ethylene oxide (EO). Material extracts were prepared using Dulbecco's Modified Eagle's Medium (DMEM) (1 g/L glucose) as the extraction vehicle at a solid‐to‐liquid ratio of 0.2 g/mL. Samples were incubated in complete medium at 37°C with agitation for 24 h (with the extraction volume adjusted to account for liquid absorption by the specimens), and the supernatants were collected as 100% (undiluted) extracts. Undiluted extracts were then diluted with complete medium to obtain 75% extracts for cell experiments. After overnight attachment, Mouse BMSCs were seeded in 12‐well plates at 5 × 10^4^ cells/well onto sterile glass coverslips. All groups were cultured under osteogenic induction conditions. Osteogenic medium consisted of DMEM (1 g/L glucose) supplemented with 10% heat‐inactivated Fetal Bovine Serum (FBS), 100 U/mL penicillin, and 100 μg/mL streptomycin (Gibco), and further supplemented with 5 mM β‐glycerophosphate, 50 μg/mL ascorbic acid 2‐phosphate, and 10 nM dexamethasone–cyclodextrin complex (water‐soluble) (Sigma‐Aldrich). Three groups were tested: Blank, GelMA extract, and GelMA+nHA + PEGDA extract. The Blank group received freshly prepared osteogenic medium, whereas the extract groups received osteogenic medium prepared using the corresponding 75% material extracts (i.e., replacing the basal medium volume with 75% extract and adding osteogenic supplements to the final concentrations stated above). Media were refreshed every other day.

### 4.9 Gene expression analysis‐qPCR

Total RNA was isolated from primary mouse BMSCs on Day 21 of osteogenic differentiation using TRI Reagent R Solution. RNA concentration and purity were assessed using a NanoDrop 1000, and Complementary DNA (cDNA) was synthesized using the TaqMan Reverse Transcription Kit. qPCR was performed on a QuantStudio 6 Flex system using TaqMan Universal Master Mix and gene‐specific assays for *ALP*, *RUNX2*, *OPN*, and *COL1A1*. *RPLP0* was used as the endogenous reference, and relative expression was calculated using the 2−ΔΔCt method. Three independent biological cultures were analyzed per group. Technical Quantitative Polymerase Chain Reaction (qPCR) duplicates were averaged before inferential analysis.

### 4.10 Protein extraction and western blotting

Cells were lysed on ice in Radioimmunoprecipitation Assay (RIPA) buffer (Beyotime, P0013B) supplemented with Phenylmethylsulfonyl Fluoride (PMSF) (final 1 mM; Beyotime, ST506) to obtain total protein. Lysates were sonicated briefly on ice and centrifuged at 15,000 × g for 6 min at 4°C, and the supernatants were collected. Protein concentration was quantified using a Bicinchoninic Acid (BCA) assay (Thermo Scientific Pierce™, 23225).

For Sodium Dodecyl Sulfate‐Polyacrylamide Gel Electrophoresis (SDS‐PAGE), 20 μg protein per lane was mixed with 5× SDS sample loading buffer (Beyotime, P0015L) and adjusted to a final loading volume of 15 μL. Proteins were separated by SDS‐PAGE and transferred onto Polyvinylidene Fluoride (PVDF) membranes (Millipore, Immobilon®‐P, IPVH00010) using a wet‐transfer system. Membranes were blocked with 5% (w/v) skimmed milk in TBST for 1 h at room temperature, incubated with primary antibodies (1:1000, overnight at 4°C) against COL1A1 (Abcam, ab34710), ALP/ALPL (R&D Systems, AF2910), RUNX2 (Cell Signaling Technology, #12556), OPN/SPP1 (Cell Signaling Technology, #88742), and GAPDH (Cell Signaling Technology, #2118), followed by Horseradish Peroxidase (HRP)‐conjugated secondary antibodies (1:3000, 1 h at room temperature). After washing in TBST (3 × 5 min), signals were developed using an HRP substrate and quantified by densitometry using ImageJ.

### 4.11 ALP staining

On Day 7, cells were washed three times with PBS and stained for ALP using a ready‐to‐use 5‐Bromo‐4‐Chloro‐3‐Indolyl Phosphate/Nitro Blue Tetrazolium (BCIP/NBT) ALP staining kit (Fixing Solution + Staining Solution; Abcam) following the manufacturer's instructions. Briefly, Fixing Solution was added to cover the cell layer and incubated for 2 min at room temperature, followed by washing with PBS/Phosphate‐Buffered Saline Containing Tween 20 (PBST). Staining Solution (ready‐to‐use) was then added (~400 μL/well) and incubated for 15–30 min at room temperature, protected from light, until a purple/blue precipitate developed. The reaction was stopped by rinsing thoroughly with PBS (or distilled water), and plates were imaged under identical acquisition settings across groups. For semi‐quantitative analysis, images were processed in ImageJ. Briefly, images were converted to 8‐bit, a consistent threshold was applied to identify ALP‐positive staining, and the ALP‐positive area fraction (%) (and/or integrated density of the thresholded signal) was measured per well using identical parameters for all groups.

### 4.12 Alizarin red staining

On Day 21, cultures were washed twice with PBS, fixed in 4% neutral buffered formalin for 15 min at room temperature, and rinsed three times with ddH_2_O. Samples were stained with 40 mM Alizarin Red S (ARS; pH 4.2, Sigma‐Aldrich) for 1 h at room temperature with gentle agitation (400 μL per well for 24‐well plates). After staining, wells were washed repeatedly with ddH_2_O until the rinse solution was clear to remove unbound dye, and plates were air‐dried prior to imaging. Images were acquired under identical illumination/exposure settings across groups. For semi‐quantitative analysis, ARS images were processed in ImageJ. Briefly, images were converted to 8‐bit (or split into Red, Green, and Blue (RGB) channels and the red channel was used), a consistent threshold was applied to segment ARS‐positive mineralized areas, and the ARS‐positive area fraction (%) (and/or integrated density of the thresholded signal) was measured per well using identical parameters for all groups.

### 4.13 Immunofluorescence staining of osteogenically differentiated BMSCs

After 21 days of osteogenic differentiation, cells cultured on sterile glass coverslips were washed three times with PBS and fixed with 4% paraformaldehyde for 30 min at room temperature. The cells were then washed with PBS and permeabilized with 0.1% Triton X‐100 in PBS for 10 min. Non‐specific binding was blocked with 3% bovine serum albumin (BSA) in PBS for 30 min at room temperature. The coverslips were incubated overnight at 4°C with primary antibodies against OCN, ALP, Prrx1, and type II collagen (COL II), typically diluted 1:100 in PBS containing 1% BSA. After three washes with PBS, the samples were incubated with the appropriate Alexa Fluor‐conjugated secondary antibodies (Invitrogen) for 1 h at room temperature in the dark, followed by three additional PBS washes. Nuclei were counterstained with Hoechst 33258 or 4’,6‐Diamidino‐2‐Phenylindole (DAPI) for 5 min at room temperature in the dark, and the coverslips were mounted using an antifade mounting medium. Fluorescence images were acquired using a Zeiss LSM800 confocal laser‐scanning microscope with identical acquisition settings across all groups, including the same objective, pinhole size, laser power, detector gain, and scanning parameters. For each sample, images were collected from at least five randomly selected fields per coverslip. Images were analyzed using ImageJ. Fluorescence channels were separated, background subtraction was applied uniformly, and a constant threshold was used to quantify the positive area fraction (%) and/or mean fluorescence intensity of OCN, ALP, and COL II. For Prrx1, nuclear masks were generated from the nuclear counterstaining channel, and nuclear Prrx1 mean fluorescence intensity per cell was quantified using identical analysis parameters across all groups.

### 4.14 Mice femoral fracture model

The 63 mice used for the in vivo study were assigned unique ear‐tag numbers and randomly allocated to the unrepaired, intramedullary implant, and BA groups using a computer‐generated random‐number sequence, with 21 mice per group. Randomization was not stratified by sex. Within each group, eight mice were used for the 2‐week terminal assessment and 13 mice for the 4‐week terminal assessment, including eight for histological analysis and five for ex vivo mechanical testing. Compatible assessments were performed sequentially on the same animals or specimens where possible. The same housing, surgical, imaging, and analytical procedures were applied across groups.

Mice were anesthetized with 1% pentobarbital sodium administered by intramuscular injection at 0.005 mL/g body weight, and adequate anesthesia was confirmed by loss of pedal withdrawal reflex. Animals were positioned in the left lateral decubitus position; the right hindlimb was shaved, disinfected, and draped. A 1 cm longitudinal lateral incision was made over the mid‐diaphysis of the right femur, and the femoral shaft was exposed by blunt dissection through the intermuscular plane while minimizing soft‐tissue disruption. To standardize the fracture site, a 1 mL syringe needle was used as a drill to create four pairs of vertically aligned perforations at the same mid‐shaft level, after which a reproducible transverse mid‐shaft fracture was generated by completing the osteotomy across the perforated segment using fine scissors. In the intramedullary fixation group, a 1 mL syringe needle was used as the intramedullary pin to stabilize the fracture. In the bone adhesive group, GelMA–nHA–PEGDA was pre‐warmed in a water bath until fully molten, applied directly onto the fracture region, molded into a circumferential circular wrap (~3 mm width) covering the fracture site, and photocrosslinked using 405 nm irradiation for 20 s in situ. In the untreated control group, no fixation or adhesive was applied. For all groups, the wound was closed layer‐by‐layer using 5–0 sutures (Ethicon).

Postoperatively, mice received analgesia and were monitored daily for wound integrity, gait, weight bearing, and general wellbeing. Fracture healing was assessed radiographically at postoperative Days 14 and 28 using consistent positioning and exposure settings. A longitudinal subset of eight mice per group underwent x‐ray imaging at both time points, and radiographs were scored independently by three experienced orthopedic surgeons using the Lane–Sandhu system. Separate terminal cohorts were euthanized at 2 or 4 weeks according to the predefined allocation. The individual mouse was the experimental unit. No animals were excluded from the analyses. x‐ray analysis included *n* = 8 mice per group, footprint analysis *n* = 6, Micro‐Computed Tomography (micro‐CT) *n* = 6, H&E histological scoring *n* = 8 per group at each time point, immunofluorescence *n* = 6, and ex vivo mechanical testing *n* = 5. These subsets overlapped where compatible and were not added together.

### 4.15 Gait analysis

Gait was assessed at postoperative Days 14 and 28 using a standard footprint test with inked paws. Mice were habituated to the apparatus before data collection and then allowed to traverse a straight runway (20 cm long × 4 cm wide) lined with a sheet of white paper placed on the floor of the track. Forepaws were coated with black ink and hind paws with red ink, consistent with commonly used footprint protocols. Each mouse was placed at one end of the runway, and a food reward was positioned at the opposite end to encourage steady forward walking; runs with stopping, turning, or obvious smearing were excluded and repeated until ≥3 usable runs per mouse were obtained. From each usable run, three to five consecutive, clearly defined hind‐paw prints were selected for analysis. The primary endpoint was hindlimb base width (base of support), defined as the mediolateral distance between the centers of the left and right hind‐paw prints for each step; values were averaged across steps and runs to obtain one value per mouse. Measurements were performed in ImageJ after spatial calibration using a ruler printed/placed in the image, with identical selection criteria applied to the intramedullary fixation, bone adhesive, and untreated control groups. Footprint analysis was performed in a predefined subset of animals (*n* = 6 per group). Multiple usable steps and runs from the same animal were averaged to generate one value per mouse; the individual mouse, rather than each footprint, was the experimental unit.

### 4.16 Micro‐CT

After euthanasia, the femoral bones of the mice were harvested and subjected to high‐resolution micro‐CT analysis using a SANCO Medical AG system 4 weeks postsurgery. The micro‐CT scans were performed with an isometric resolution of 10 μm. Prior to scanning, the bone fracture samples were fixed in a 4% paraformaldehyde solution overnight to preserve their structural integrity. During the micro‐CT scans, the following parameters were used: a voltage of 70 kV, a current of 112 μA, and a slice thickness of 0.048 mm. The scans were conducted in medium‐resolution mode, utilizing a reconstruction matrix of 1024 and an integration time of 200 ms. To analyze the bone structure, threshold values for bone were set within the range of 315–543 Hounsfield units. To determine the volume of interest for analysis, a circular contour with a diameter of 10 mm was drawn around the center of each bone defect. Subsequently, various bone parameters, including BV, BV fraction (BV/TV ratio), Tb.N, Tb.Th, and trabecular separation (Tb.Sp), were quantitatively analyzed according to established protocols. Ex vivo micro‐CT was performed on a predefined subset of harvested specimens (*n* = 6 per group), as only intact specimens with sufficient image quality could be reliably analyzed.

### 4.17 Hematoxylin and eosin staining

After fixation, Ethylenediaminetetraacetic Acid (EDTA) decalcification, paraffin embedding, and sectioning (4 μm), paraffin sections were subjected to H&E staining to evaluate callus morphology and tissue organization. Briefly, sections were deparaffinized in xylene (2 × 10 min) and rehydrated through a graded ethanol series (100%, 95%, 80%, 70%, 5 min each) to distilled water. Sections were stained with hematoxylin for 5 min, rinsed in running tap water, and differentiated in 1% acid alcohol for 10 s, followed by blueing in 0.2% ammonia water for 60 s. Sections were then counterstained with eosin for 2 min, dehydrated through ascending ethanol (70%–100%), cleared in xylene (2 × 5 min), and mounted with a resinous mounting medium. Stained sections were imaged under bright‐field microscopy using identical acquisition settings across groups. For semi‐quantitative assessment, H&E sections spanning the fracture region were independently scored by three experienced orthopedic surgeons blinded to group allocation using the Menendi et al.^38^ 1–10 callus maturity scale (from fibrous tissue to mature bone), with mean scores used for analysis, and new bone area within the callus was quantified by planimetric analysis^39^ in ImageJ within a predefined ROI and expressed as New Bone Area (NBA)/Region of Interest (ROI) (%), with measurements independently performed and cross‐checked by the same three blinded assessors. the mean value of the three assessments was used for downstream analysis. After H&E staining, histological scoring was performed in *n* = 8 independent mice per group at each time point. New bone area was quantified in a predefined subset of *n* = 3 independent mice per group at each time point with a consistent sectioning plane and clearly delineated callus. Multiple fields or assessor scores from the same specimen were averaged to yield one value per mouse before statistical analysis.

### 4.18 Immunohistochemical evaluation

Mouse femora were fixed in 4% paraformaldehyde for 24 h and decalcified in 10% EDTA at 4°C. After cryoprotection, specimens were embedded in Optimal Cutting Temperature (OCT) compound and cryosectioned for immunofluorescence analysis. Sections were permeabilized, blocked, and incubated with primary antibodies against ALP/ALPL, OCN, Prrx1, and COLII, followed by species‐matched fluorescent secondary antibodies and DAPI counterstaining. Images were acquired using identical confocal settings across groups. For ALP, OCN, and Prrx1, marker‐positive cells were expressed relative to total DAPI‐positive nuclei. COLII was quantified as fluorescence intensity within predefined callus regions of interest. Six independent mice were analyzed per group.

### 4.19 Statistical analysis

Data are presented as mean ± standard deviation (SD) and were analyzed in GraphPad Prism 9.0. The experimental unit was the independent material specimen, independent cell culture, or individual mouse, as specified in each figure legend. Technical repeats and multiple fields or measurements from the same experimental unit were averaged before inferential analysis. Normality and homogeneity of variance were assessed using the Shapiro–Wilk and Brown–Forsythe tests, respectively. Longitudinal x‐ray and gait data obtained from the same mice were analyzed using two‐way repeated‐measures Analysis of Variance (ANOVA). Quantitative H&E data obtained from different mice at 2 and 4 weeks were analyzed using ordinary two‐way ANOVA. Tukey's multiple‐comparisons test was used for treatment‐group comparisons within each time point, and Šídák's multiple‐comparisons test was used for time‐point comparisons within each group. Other data sets involving multiple independent groups were analyzed using one‐way ANOVA followed by Tukey's multiple‐comparisons test. Exact sample sizes are provided in the figure legends, and exact p values are reported in the figures where appropriate. Two‐sided *p* < 0.05 was considered statistically significant.

## AUTHOR CONTRIBUTIONS

**Yuqi Liu**: Conceptualization; methodology; investigation; formal analysis; data curation; software; validation; visualization; funding acquisition; writing—original draft. **Hao Luo**: Methodology; investigation; data curation. **Bin Guo**: Methodology; investigation; data curation. **Xiangming Chen**: Methodology; investigation; data curation. **Martin J. Stoddart**: Supervision; project administration; visualization; writing—review and editing. **Wen Chen**: Supervision; writing—review and editing. **Xucheng Yang**: Supervision; writing—review and editing. **Yong Zhu**: Supervision; resources; project administration; writing—review and editing. **Jianxi Zhu**: Supervision; funding acquisition; resources; project administration; writing—review and editing.

## CONFLICT OF INTEREST STATEMENT

The authors declare no conflict of interest.

## Data Availability

The data that support the findings of this study are available from the corresponding author upon reasonable request.
